# Correction: Cai et al. Sex Reversal Induced by Dietary Supplementation with 17α-Methyltestosterone during the Critical Period of Sex Differentiation in Oriental River Prawn (*Macrobrachium nipponense*). *Animals* 2023, *13*, 1369

**DOI:** 10.3390/ani13152489

**Published:** 2023-08-01

**Authors:** Pengfei Cai, Huwei Yuan, Zijian Gao, Peter Daka, Hui Qiao, Wenyi Zhang, Sufei Jiang, Yiwei Xiong, Yongsheng Gong, Yan Wu, Shubo Jin, Hongtuo Fu

**Affiliations:** 1 Wuxi Fisheries College, Nanjing Agricultural University, Wuxi 214081, China; ckgg5436@126.com (P.C.); yuan08102021@126.com (H.Y.); gaozijiangenomics@163.com (Z.G.); peterdaka1@yahoo.com (P.D.); 2 Key Laboratory of Freshwater Fisheries and Germplasm Resources Utilization, Ministry of Agriculture and Rural Affairs, Freshwater Fisheries Research Center, Chinese Academy of Fishery Sciences, Wuxi 214081, China; qiaoh@ffrc.cn (H.Q.); zhangwy@ffrc.cn (W.Z.); jiangsf@ffrc.cn (S.J.); xiongyw@ffrc.cn (Y.X.); gongys@ffrc.cn (Y.G.); wuy@ffrc.cn (Y.W.)

## Error in Table

In the original publication [1], there was a mistake in Table 3, entitled “Sequences of primers used”. The primer sequence of EIF (EIF-F and EIF-R) was incorrectly entered. The corrected Table 3 appears below. The authors state that the scientific conclusions are unaffected. This correction was approved by the Academic Editor. The original publication has also been updated.

## Figures and Tables

**Table 3:** Primers of sequence used.

Primer Name	Sequence (5′ → 3′)	Description
*EIF*-F	CATGGATGTACCTGTGGTGAAAC	FWD primer for *EIF* expression
*EIF*-R	CTGTCAGCAGAAGGTCCTCATTA	RVS primer for *EIF* expression
*Vg*-F	GAAGTTAGCGGAGATCTGAGGT	FWD primer for *Vg* expression
*Vg*-R	CCTCGTTGACCAATCTTGAGAG	RVS primer for *Vg* expression
*Vgr*-F	ACCACTCGGATGAGGACGACT	FWD primer for *Vgr* expression
*Vgr*-R	CCATCTTTGCACTGGTAGTGGT	RVS primer for *Vgr* expression
*IAG*-F	CGCCTCCGTCTGCCTGAGATAC	FWD primer for *IAG* expression
*IAG*-R	CCTCCTCCTCCACCTTCAATGC	RVS primer for *IAG* expression
*SG*-F	ACCCTAGCCCCAGTACGTGTT	FWD primer for *SG* expression
*SG*-R	AGAGGTGGTGAAGCTGTCTCTCA	RVS primer for *SG* expression
*DMRT*-F	ACGACCTTAGTAGGATGGACAGT	FWD primer for *DMRT* expression
*DMRT*-R	GAGTGGAGGCAATAGAATGGGTA	RVS primer for *DMRT* expression
*Foxl2*-F	AAATCCCTGTACGACCACATCG	FWD primer for *Foxl2* expression
*Foxl2*-R	CTTCGTCGGGTAGAGCATCTCC	RVS primer for *Foxl2* expression
*SoxE*-F	ACATAGATCGCGCAGAAATGAAC	FWD primer for *SoxE* expression
*SoxE*-R	CCAAGGAAGGAAGACTTGTGAGT	RVS primer for *SoxE* expression

## References

[1] Cai P., Yuan H., Gao Z., Daka P., Qiao H., Zhang W., Jiang S., Xiong Y., Gong Y., Wu Y. (2023). Sex Reversal Induced by Dietary Supplementation with 17α-Methyltestosterone during the Critical Period of Sex Differentiation in Oriental River Prawn (*Macrobrachium nipponense*). Animals.

